# Optimization Methods for Radon Progeny Measurement Based on the Three-Stage Method

**DOI:** 10.3390/toxics13010031

**Published:** 2025-01-02

**Authors:** Xiangming Cai, Xuwei Zeng, Fengdi Qin, Jian Shan

**Affiliations:** 1 School of Nuclear Science and Technology, University of South China, Hengyang 421001, China; cxm9165@163.com (X.C.); 17356022492@163.com (X.Z.); 2 Radon Key Laboratory of Hunan Province, Hengyang 421001, China; 3 China Atomic Energy Science Research Institute, National Atomic Energy Anti-Radiation Center, Beijing 102413, China; 20202010110573@stu.usc.edu.cn

**Keywords:** radon progeny, three-stage method for radon measurement, time optimization

## Abstract

In different measurement tasks, the duration allocated for radon progeny concentration measurement varies, and the requirements for measurement precision also differ. To accommodate the needs of various radon progeny concentration measurement tasks, this study takes the error in radon progeny concentration measurement as the optimization goal and the time points of the three-stage method as the optimization target, establishing an optimized three-stage method for radon progeny concentration measurement. The optimization algorithm allocates the three time periods under any total measurement time requirement, ensuring the highest measurement precision. The optimization algorithm can also optimize the measurement errors for ^218^Po, ^214^Pb, and ^214^Bi according to task requirements. By comparing with existing three-stage methods, when the total measurement time is 30 min, the optimization results are similar to Thomas’s three-stage method; when the total measurement time is 15 min, the measurement results are consistent with the rapid three-stage method. Therefore, the optimized results of the three-stage method are reasonable and reliable, providing technical support for different radon progeny measurement tasks.

## 1. Introduction

Radon is widely found in soil, building materials, natural gas, and other substances. The radiation dose contributed by radon and its progeny accounts for more than 52% of the natural radiation dose, making it a significant source of natural radiation [1]. High radon environments are particularly common in settings such as mines, kilns, and basements [2]. When inhaled by humans, radon decays to produce progeny that can cause internal exposure, posing a threat to human health [3]. The dose contributed by the inhalation of radon and its progeny mainly comes from short-lived radon progeny [4]. Therefore, to limit the personal dose of workers, it is necessary to accurately measure the concentration of radon progeny in the environment.

The earliest process for measuring radon progeny concentration involved sampling for 5 min and then recording the alpha total counts at 5, 15, and 30 min, respectively, followed by analytical calculations to determine the concentration of radon progeny (i.e., the three-point method) [5]. Due to its simplicity, this method was widely applied after being proposed by Tsivoglou in 1953 [6], but it was not highly accurate. In 1972, Thomas replaced the count rate at specific times with the total counts over different time periods, establishing the famous Thomas three-stage method [7]. Theoretically, the time selection for the three-stage method is arbitrary, but different choices result in different measurement errors. Thomas calculated and compared errors from 324 sets of data to determine the measurement time periods for the three-stage method (2 to 5 min, 6 to 20 min, and 21 to 30 min) to calculate the concentrations of three radon progeny [7]. Additionally, the Lavender three-stage method is more suitable for the measurement of unattached radon progeny [8]. The Lavender three-stage method can measure two samples simultaneously, with higher sensitivity for ^218^Po, ^214^Pb, and ^214^Bi measurements [8]. However, the Lavender three-stage method requires a 15-min sampling period, which is time-consuming and cumbersome. The Scott three-stage method has a measurement time of up to 90 min, and by reducing the contribution of ^218^Po during the measurement process, it has a smaller error compared to others [9].

The alpha spectrometry method involves an alpha spectrometer to measure the count rates of ^218^Po and ^214^Bi to calculate the concentration of radon progeny [5] (In alpha spectrometry, the primary measurement is the count of alpha particles, specifically RaA (^218^Po) and RaC (^214^Bi). However, due to the rapid decay of RaC, the counts for RaC’ (^214^Po) are typically included in the count for RaC). The alpha spectrometry method has lower measurement errors than the three-point method, but the process requires the involvement of an alpha spectrometer, which is highly inconvenient [10]. Therefore, the alpha spectrometry method is not often considered for measuring radon progeny concentrations in environments such as the wild, underground tunnels, etc. During measurement, image information from the IP imaging plate is obtained using the Typhoon 7000 device, requiring researchers to identify and count the alpha tracks in the images themselves, and then retro-calculate the concentration of radon progeny using methods such as the Thomas three-stage method [11,12,13]. Chen Bo and others analyzed the track decay effect of the IP imaging plate [14], Qin Fengdi and others corrected the overlap of alpha tracks in the IP [15], and proposed a method using convolutional neural networks to read the number of alpha particles [16]. Therefore, portable measurement methods represented by IP have become a popular means of radon progeny measurement [17].

Measurement methods represented by IP mainly use the three-stage method [17,18]. However, the requirements for measurement sensitivity vary depending on the task. For example, scientific research requires high measurement accuracy, while field environmental measurements require relatively lower accuracy. In addition, different tasks allow for different measurement times. Longer measurement times can greatly improve measurement accuracy. Optimizing the three measurement time periods within a short measurement time can also greatly improve measurement accuracy (such as the choice of time for the Thomas three-stage method). Therefore, it is very necessary to establish a method that optimizes different time periods in the three-stage method under the condition of a determined total measurement time, ensuring that the measurement accuracy is minimized under that condition.

This paper, based on the decay process of radon progeny on the filter membrane before and after sampling, conducts a study on the relationship between error and measurement time. With error as the optimization goal and measurement time periods as the optimization object, it fully considers actual measurement requirements and proposes an optimized three-stage method for measuring radon progeny concentrations, ensuring that the measurement error can be minimized when the total measurement time is determined.

## 2. Method

### 2.1. Sampling of Radon Progeny

**Figure 1:**
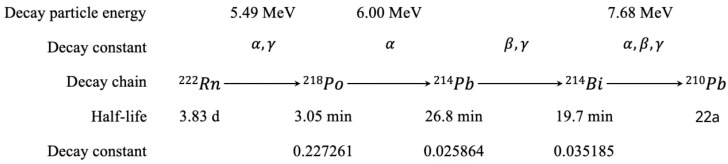
Radon Progeny and Their Decay Scheme.

When a mixture of radon and its progeny passes through a filter membrane, the progeny are collected by the filter. The number of radon progeny on the membrane increases due to collection but simultaneously decreases due to decay (Figure 1). Therefore, during the radon progeny sampling process, the change in their numbers is as follows:

(1) $$\left\{\begin{array}{c} \frac{dN_{2}\left(t\right)}{dt}=\eta \theta_{2}v-\lambda_{2}N_{2}\left(t\right)\;\;\;\;\;\;\;\;\;\;\;\;\;\;\;\;\;\;\;\;\\ \frac{dN_{3}\left(t\right)}{dt}=\eta \theta_{3}v+\lambda_{2}N_{2}\left(t\right)-\lambda_{3}N_{3}\left(t\right)\\ \frac{dN_{4}\left(t\right)}{dt}=\eta \theta_{4}v+\lambda_{3}N_{3}\left(t\right)-\lambda_{4}N_{4}\left(t\right) \end{array}\right.$$

where $N_{i}\left(t\right)$ is the number of atoms of the i-th progeny on the filter membrane at the sampling time t; η is the filtration efficiency of the filter membrane; v is the sampling flow rate; $\lambda_{i}$ is the decay constant of the i-th progeny; and $\theta_{i}$ is the atomic concentration of the i-th progeny.

Typically, in experimental or field testing procedures, a duration of 5 min is selected for sampling. Consequently, considering the solution to the kinetic equations (Equation (1)), The process of variation in the number of radon progeny atoms during sampling can be described as follows (Appendix A):

(2) $$\left\{\begin{array}{c} N_{2}\left(t\right)=2.988\cdot \theta_{2}\\ N_{3}\left(t\right)=1.921\cdot \theta_{2}+4.69\cdot \theta_{3}\\ N_{4}\left(t\right)=0.08707\cdot \theta_{2}+0.2922\cdot \theta_{3}+4.585\cdot \theta_{4} \end{array}\right.$$

To convert the atomic concentration of radon progeny (θ) into radioactive activity concentration (C=λθ)), the calculation method for the concentration of radon progeny is as follows:

(3) $$\left\{\begin{array}{c} C_{2}=0.07606\cdot N_{2}\left(t\right)\\ C_{3}=0.005515\cdot N_{3}\left(t\right)-0.003545\cdot N_{2}\left(t\right)\\ C_{4}=8.376\times 10^{-5}\cdot N_{2}\left(t\right)-0.0004781\cdot N_{3}\left(t\right)+0.007674 \end{array}\right.$$

where $C_{i}$ is the radioactive concentration of the i-th progeny.

### 2.2. Changes in Radon Progeny on the Filter Membrane

After sampling is completed, the radon progeny collected on the membrane (^218^Po, ^214^Bi, ^210^Pb) will undergo a series of changes through cascade decay. Each step in the cascade decay generates radiation particles, but only the decay of ^218^Po and ^218^Bi is accompanied by the emission of alpha particles. The three-step method uses these changes in the number of alpha particles to establish a system of equations for analyzing the concentrations of different radon progeny.

After the sampling is completed, the number of different radon progeny on the filter membrane can be described as a function of time T using the kinetic equation (Equation (4)):

(4) $$\left\{\begin{array}{c} \;\frac{dN_{2}\left(T\right)}{dT}=-\lambda_{2}N_{2}\left(T\right)\;\;\;\;\;\;\;\;\;\;\;\;\;\;\;\;\;\;\;\\ \frac{dN_{3}\left(T\right)}{dT}=\lambda_{2}N_{2}\left(T\right)-\lambda_{3}N_{3}\left(T\right)\;\\ \frac{dN_{4}\left(T\right)}{dT}=\lambda_{3}N_{3}\left(T\right)-\lambda_{4}N_{4}\left(T\right) \end{array}\right.$$

where $N_{i}\left(T\right)$ is the number of atoms of the i-th species that have decayed to time T. “t” stands for the sampling time, while “T” indicates the time starting from the end of the sampling period until the measurement period concludes.

(5) $$N_{2}\left(T\right)=\exp\left(-0.2273\cdot T\right)\cdot N_{2}\left(t\right)\;\;\;\;\;\;\;\;\;\;\;\;\;\;\;\;\;\;\;\;\;\;\;\;\;\;\;\;\;\;\;\;\;\;\;\;\;\;\;\;\;\;\;\;\;\;\;\;\;\;\;\;\;\;\;\;\;\;\;\;\;\;\;\;\;\;\;\;\;\;\;\;\;\;\;\;\;\;N_{3}\left(T\right)=\exp\left(-0.02586\cdot T\right)\cdot N_{3}\left(t\right)+N_{2}\left(t\right)\cdot \left(1.128\cdot \exp\left(-0.02586\cdot T\right)-1.128\cdot \exp\left(-0.2273\cdot T\right)\right)\;N_{4}\left(T\right)=N_{2}\left(t\right)\cdot \left(3.131\cdot \exp\left(-0.02586\cdot T\right)-3.283\cdot \exp\left(-0.03518\cdot T\right)+0.1519\cdot \exp\left(-0.2273\cdot T\right)\right)\;+\exp\left(-0.03518\cdot T\right)\cdot N_{4}\left(t\right)-N_{3}\left(t\right)\cdot \left(2.775\cdot \exp\left(-0.03518\cdot T\right)-2.775\cdot \exp\left(-0.02586\cdot T\right)\right)$$

After the sampling is completed, the initial value for the cascade decay of radon progeny is the amount deposited at the last moment of sampling. Therefore, the total alpha activity ($I\left(\alpha \right)$) of ^218^Po and ^214^Bi on the filter membrane at time T is:

(6) $$I\left(\alpha \right)=\lambda_{2}N_{2}\left(T\right)+\lambda_{4}N_{4}\left(T\right)$$

Following the completion of sampling, within the time intervals T_1_ to T_2_, T_3_ to T_4_, and T_5_ to T_6_, the number of alpha particles produced by the decay of radon progeny is as follows:

(7) $$\begin{array}{cc} I\left(T_{1},T_{2}\right)=N_{4}\left(t\right) & \cdot \left(1.0\cdot \exp\left(-0.03518\cdot T_{1}\right)-1.0\cdot \exp\left(-0.03518\cdot T_{2}\right)\right)-N_{2}(t)\\ & \cdot (3.283\cdot \exp\left(-0.03518\cdot T_{1}\right)-3.283\cdot \exp\left(-0.03518\cdot T_{2}\right)-4.26\cdot \exp\left(-0.02586\cdot T_{1}\right)+4.26\\ & \cdot \exp\left(-0.02586\cdot T_{2}\right)-1.024\cdot \exp\left(-0.2273\cdot T_{1}\right)+1.024\cdot \exp\left(-0.2273\cdot T_{2}\right))-N_{3}(t)\\ & \cdot (2.775\cdot \exp\left(-0.03518\cdot T_{1}\right)-2.775\cdot \exp\left(-0.03518\cdot T_{2}\right)-3.775\cdot \exp\left(-0.02586\cdot T_{1}\right)\\ & +3.775\cdot \exp\left(-0.02586\cdot T_{2}\right)) \end{array}$$

(8) $$\begin{array}{cc} I\left(T_{3},T_{4}\right)=N_{4}\left(t\right) & \cdot \left(1.0\cdot \exp\left(-0.03518\cdot T_{3}\right)-1.0\cdot \exp\left(-0.03518\cdot T_{4}\right)\right)-N_{2}(t)\\ & \cdot (3.283\cdot \exp\left(-0.03518\cdot T_{3}\right)-3.283\cdot \exp\left(-0.03518\cdot T_{4}\right)-4.26\cdot \exp\left(-0.02586\cdot T_{3}\right)+4.26\\ & \cdot \exp\left(-0.02586\cdot T_{4}\right)-1.024\cdot \exp\left(-0.2273\cdot T_{3}\right)+1.024\cdot \exp\left(-0.2273\cdot T_{4}\right))-N_{3}(t)\\ & \cdot (2.775\cdot \exp\left(-0.03518\cdot T_{3}\right)-2.775\cdot \exp\left(-0.03518\cdot T_{4}\right)-3.775\cdot \exp\left(-0.02586\cdot T_{3}\right)\\ & +3.775\cdot \exp\left(-0.02586\cdot T_{4}\right)) \end{array}$$

(9) $$\begin{array}{c} I\left(T_{5},T_{6}\right)=N_{4}\left(t\right)\cdot \left(1.0\cdot \exp\left(-0.03518\cdot T_{5}\right)-1.0\cdot \exp\left(-0.03518\cdot T_{6}\right)\right)-\\ N_{2}(t)\cdot (3.283\cdot \exp\left(-0.03518\cdot T_{5}\right)-3.283\cdot \exp\left(-0.03518\cdot T_{6}\right)-4.26\cdot \exp\left(-0.02586\cdot T_{5}\right)+4.26\cdot \\ \exp\left(-0.02586\cdot T_{6}\right)-1.024\cdot \exp\left(-0.2273\cdot T_{5}\right)+1.024\cdot \exp\left(-0.2273\cdot T_{6}\right))-N_{3}(t)\cdot (2.775\cdot \\ \exp\left(-0.03518\cdot T_{5}\right)-2.775\cdot \exp\left(-0.03518\cdot T_{6}\right)-3.775\cdot \exp\left(-0.02586\cdot T_{5}\right)+3.775\cdot \exp\left(-0.02586\cdot T_{6}\right)) \end{array}$$

Based on the total number of alpha particles during the intervals T_1_-T_2_, T_3_-T_4_, and T_5_-T_6_, it is possible to determine the initial atomic counts of the three types of radon progeny and substitute them into Equation (2) to obtain the calculation method for the concentration of radon progeny. Since the formula contains unknown time variables and is excessively lengthy, only the mapping relationships are presented here. The mapping relationship formula for calculating the concentration of radon progeny is as follows:

(10) $$\left\{\begin{array}{c} C_{2}=f_{C_{2}}(I\left(T_{1},T_{2}\right),I\left(T_{3},T_{4}\right),I\left(T_{5}5,T_{6}\right),T_{1},T_{2},T_{3},T_{4},T_{5},T_{6},R)\\ C_{3}=f_{C_{3}}(I\left(T_{1},T_{2}\right),I\left(T_{3},T_{4}\right),I\left(T_{5}5,T_{6}\right),T_{1},T_{2},T_{3},T_{4},T_{5},T_{6},R)\\ C_{4}=f_{C_{4}}(I\left(T_{1},T_{2}\right),I\left(T_{3},T_{4}\right),I\left(T_{5}5,T_{6}\right),T_{1},T_{2},T_{3},T_{4},T_{5},T_{6},R) \end{array}\right.$$

However, in the actual testing process, the relationship between the decay count of radon progeny and the instrument’s count is as follows (E represents the detection efficiency of the instrument, while $K_{a}$ absorption factor of the filter):

(11) $$\left\{\begin{array}{c} I\left(T_{1},T_{2}\right)=\frac{1}{E\cdot K_{a}}\left(G\left(T_{1},T_{2}\right)-\left(T_{2}-T_{1}\right)\cdot R\right);\\ I\left(T_{3},T_{4}\right)=\frac{1}{E\cdot K_{a}}\left(G\left(T_{3},T_{4}\right)-\left(T_{4}-T_{3}\right)\cdot R\right);\\ I\left(T_{5},T_{6}\right)=\frac{1}{E\cdot K_{a}}\left(G\left(T_{5},T_{6}\right)-\left(T_{6}-T_{5}\right)\cdot R\right); \end{array}\right.$$

where $G\left(T_{1},T_{2}\right)$, $G\left(T_{3},T_{4}\right)$, and $G\left(T_{5},T_{6}\right)$ represent the instrument counts during the time intervals T_1_ − T_2_, T_3_ − T_4_, and T_5_ − T_6_, respectively.

Therefore, by substituting Equation (12) into Equation (11), the method for calculating the concentration of radon progeny that takes into account the background count can be obtained.

(12) $$\left\{\begin{array}{c} C_{2}=f_{C_{2}}(G\left(T_{1},T_{2}\right),G\left(T_{3},T_{4}\right),G\left(T_{5},T_{6}\right),T_{1},T_{2},T_{3},T_{4},T_{5},T_{6},R)\\ C_{3}=f_{C_{3}}(G\left(T_{1},T_{2}\right),G\left(T_{3},T_{4}\right),G\left(T_{5},T_{6}\right),T_{1},T_{2},T_{3},T_{4},T_{5},T_{6},R)\\ C_{4}=f_{C_{4}}(G\left(T_{1},T_{2}\right),G\left(T_{3},T_{4}\right),G\left(T_{5},T_{6}\right),T_{1},T_{2},T_{3},T_{4},T_{5},T_{6},R) \end{array}\right.$$

### 2.3. Error Analysis

Uncertainty arises from both objective and subjective measurement errors, representing the difference between the measured result of an object and its true value. Therefore, during the testing process, errors will occur due to uncertainty. Minimizing these errors is crucial for accurate radon progeny concentration measurements. In Equation (10), the variables v, E, η (eta), and K_α_ all have associated uncertainties. However, these errors are determined during the sampling phase and are thus established when allocating the optimized time segments. Consequently, the parameters v, E, η (eta), and K_α_ and their errors do not affect the allocation of the three time segments in the optimization process. Therefore, these parameters are set to 1 in the study. Additionally, the counting of alpha particles during different time periods G(T_1_, T_2_), G(T_3_, T_4_), and G(T_5_, T_6_) will also have errors, as will the background measurements. According to the principles of error propagation, the relationship between the concentrations of the three radon progeny and the measurement errors is as follows:

(13) $$\begin{array}{r} \sigma_{2}=({\left(\frac{\partial C_{2}}{\partial G(T_{1},T_{2})}\sqrt{G(T_{1},T_{2})}\right)}^{2}+{\left(\frac{\partial C_{2}}{\partial G(T_{3},T_{4})}\sqrt{G(T_{3},T_{4})}\right)}^{2}\\ +{\left(\frac{\partial C_{2}}{\partial G(T_{5},T_{6})}\sqrt{G(T_{5},T_{6})}\right)}^{2}+{\left(\frac{\partial C_{2}}{\partial R}\sqrt{R}\right)}^{2}{)}^{\frac{1}{2}} \end{array}$$

(14) $$\begin{array}{r} \sigma_{3}=({\left(\frac{\partial C_{3}}{\partial G(T_{1},T_{2})}\sqrt{G(T_{1},T_{2})}\right)}^{2}+{\left(\frac{\partial C_{3}}{\partial G(T_{3},T_{4})}\sqrt{G(T_{3},T_{4})}\right)}^{2}\\ +{\left(\frac{\partial C_{3}}{\partial G(T_{5},T_{6})}\sqrt{G(T_{5},T_{6})}\right)}^{2}+{\left(\frac{\partial C_{3}}{\partial R}\sqrt{R}\right)}^{2}{)}^{\frac{1}{2}} \end{array}$$

(15) $$\begin{array}{r} \sigma_{4}=({\left(\frac{\partial C_{4}}{\partial G(T_{1},T_{2})}\sqrt{G(T_{1},T_{2})}\right)}^{2}+{\left(\frac{\partial C_{4}}{\partial G(T_{3},T_{4})}\sqrt{G(T_{3},T_{4})}\right)}^{2}\\ +{\left(\frac{\partial C_{4}}{\partial G(T_{5},T_{6})}\sqrt{G(T_{5},T_{6})}\right)}^{2}+{\left(\frac{\partial C_{4}}{\partial R}\sqrt{R}\right)}^{2}{)}^{\frac{1}{2}} \end{array}$$

where $\sigma_{i}$ represents the measurement error for the i-th progeny.

Background measurements are typically conducted before sampling the specimens. During the sample measurement, both the background count and the errors introduced by the local measurement do not affect the allocation of time in the three-step method. In this study’s optimization process, the background is set to zero.

However, in the optimization, one can choose to target the deviation of a specific radon progeny or aim to minimize the sum of all errors ($\sigma_{T}$) as the optimization goal.

(16) $$\sigma_{T}=\sigma_{2}+\sigma_{3}+\sigma_{4}$$

### 2.4. Optimization Method for Radon Progeny Measurement Using the Three-Step Approach

According to Equations (14)–(16), the error in measuring radon progeny concentrations using the three-step method is related to the distribution of measurement time. Consequently, this paper develops an optimization algorithm to optimize the measurement times of the three-step method to ensure minimal error.

The study targets the radon progeny error as the optimization goal, with the six measurement time points in the three-step method as the subjects of optimization, utilizing the fmincon function for this optimization. Typically, a gap of more than 2 min is required from the completion of sampling to the commencement of measurement, thus setting the lower limit of measurement time to 2 min. Depending on different measurement task requirements, the total measurement time varies. Therefore, the upper limit for the optimization of all time points is set to the total measurement time. Since the algorithm requires initial values to proceed with optimization, the study equally divides the time from 2 min to the total measurement time into six time points as the initial values for these six time points for optimization. To allow sufficient time for operations, the interval between two measurement periods should be more than 1 min, and T_6_ should be the total measurement time. Therefore, during the optimization process, the constraints can be set as follows:

### 2.5. Radon Progeny Measurement Procedure

This study proposes a method for measuring radon progeny concentrations that ensures the measurement errors are minimized. To ensure the reliability of the measurement process, the following steps are required:

1. Before measuring radon progeny concentrations, the total measurement time must first be determined;
2. The measurement time segments are then established through an optimization algorithm;
3. Sampling is conducted at a flow rate of v (L/min) for 5 min;
4. After sampling is completed, the alpha counts of the samples during the three time segments are measured, respectively denoted as G(T_1_,T_2_), G(T_3_,T_4_), G(T_5_,T_6_);
5. The radon progeny concentrations are calculated using Equation (11);
6. The standard deviation is computed using Equation (12).

## 3. Results

### 3.1. Optimization Algorithm Performance

**Figure 2:**
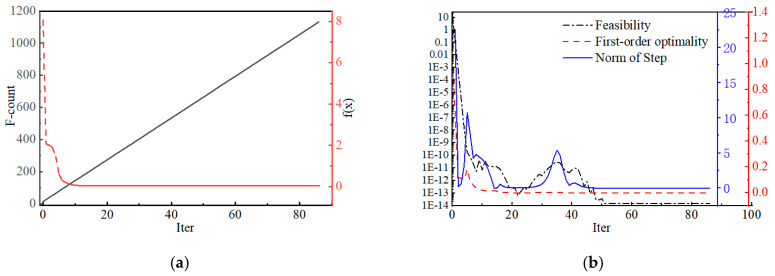
Optimization process of fmincon function.

In the optimization process described above, the performance of the “fmincon” [19] function can be assessed by several key metrics. Figure 2a is F-count and f(x) in the optimization process; Figure 2b is the Feasibility, First-order optimality, and Norm of step.

1. F-count: This is the number of function evaluations, that is, the number of times the objective function is computed. As iterations proceed, the F-count gradually increases, indicating that the algorithm is exploring the solution space to find the optimal solution (Figure 2a).
2. f(x): This is the value of the objective function. From the output results, it can be seen that the objective function value rapidly decreases from the initial 8.06 × 10^0^ and then stabilizes in subsequent iterations, ultimately reaching a very small value (such as 1.42 × 10^−14^). This indicates that the algorithm effectively reduces the objective function value, approaching the optimal solution (Figure 2a).
3. Feasibility: This metric measures the feasibility of the current solution, i.e., whether the solution meets all constraints. During the iteration process, the Feasibility value gradually decreases, indicating that the solution is increasingly close to satisfying all the constraint conditions (Figure 2b).
4. First-order optimality: This indicator measures the first-order optimality of the solution, that is, the norm of the gradient. A decrease in the first-order optimality metric suggests that the algorithm is approaching a point where the gradient is zero, which is a characteristic of a local optimal solution (Figure 2b).
5. Norm of step: This is the norm of the solution update step size in each iteration. The size of the step reflects the distance the algorithm moves in the solution space. As iterations proceed, the step size gradually decreases, indicating that the algorithm is refining its search to more precisely locate the optimal solution (Figure 2b).

From these metrics, it can be seen that the “fmincon” function exhibits good convergence during the optimization process. The significant decrease in the objective function value and the first-order optimality metric indicate that the algorithm effectively finds a solution close to optimal. The improvement in Feasibility indicates that the solution increasingly meets the constraint conditions. However, it should be noted that although these metrics suggest the algorithm is approaching an optimal solution, whether the final solution is globally optimal requires further verification. Additionally, minor fluctuations in the step size and first-order optimality metrics during the optimization process may be due to numerical stability issues when the algorithm handles nonlinear constraints. In practical applications, it may be necessary to adjust the algorithm parameters to improve the quality of the solution and the robustness of the algorithm.

### 3.2. The Three-Step Method with Different Total Measurement Times

Depending on the task at hand, the total time allowed for measuring radon progeny concentrations may not be the same. This study analyzed how the distribution of the three measurement time segments within the three-step method varies under different measurement durations, providing vital data support for experiments and environmental measurements. Among the three time segments, as time increases, the duration of each segment also increases, but the segment from T_5_ to T_6_ increases the fastest, while the segment from T_1_ to T_2_ increases the slowest. Under the set conditions, the time interval between two measurements must be more than 1 min (that is, T_3_ to T_2_ > 1 min, T_5_ to T_4_ > 1 min), and in the optimization results, the time intervals from T_3_ to T_2_ and from T_5_ to T_4_ are both 1 min. Thus, the optimization results meet the experimental operational requirements and also indicate that during the optimization process, as much time as possible was allocated to the measurement segments.

**Figure 3:**
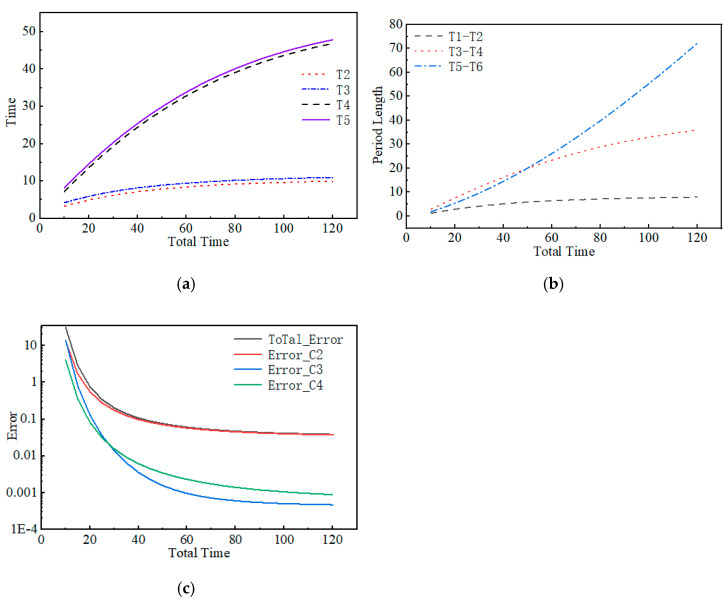
(**a**) is the time distribution of the three-step method, (**b**) is the length of time, respectively, and (**c**) is the measurement error.

Furthermore, the study found that as the measurement time increases, the sum of all radon progeny deviations and the measurement deviations of the three progeny decrease, and the sum of errors shows an inflection point around 30 min (that is, the error decline gradually slows down). Therefore, 30 min as the total measurement time can meet most measurement needs while reducing time waste. The optimization goal is to ensure the deviations of the three radon progeny reach a minimum, without considering the measurement deviation of a single progeny, thus further reducing the measurement deviation of different progeny is needed to meet various measurement tasks. As shown in Figure 3.

### 3.3. Optimizing with Different Radon Progeny Errors as Targets

During the radon progeny measurement process, it is necessary to ensure that deviations are minimized to achieve the highest possible testing accuracy. The three-step method proposed in this study can reduce measurement bias by reasonably allocating the three measurement time segments. However, in the aforementioned research, optimizing with the sum of progeny errors as the target does not meet the need to reduce the bias of different radon progeny. Therefore, it is possible to optimize the time segments by targeting different biases separately, ensuring the satisfaction of various measurement requirements.

This study uses 30 min as the total measurement time and conducts optimizations of the three-step method with different radon progeny biases as optimization targets, with the results shown in the table. The research findings indicate that when different radon progeny biases or combinations are used as optimization targets, there is a noticeable change in the measurement times of the three-step method (Table 1). Therefore, based on actual needs, during the measurement process, the appropriate duration of the three segments should be chosen according to requirements.

**Figure 4:**
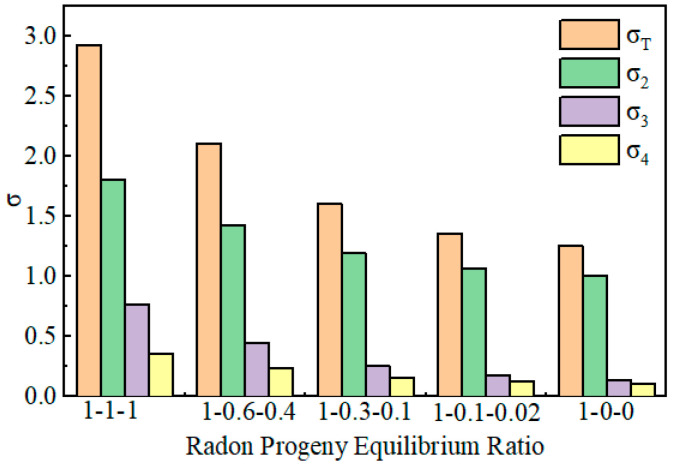
Errors Corresponding to the Optimization Results for Different Radon Daughter Equilibrium Ratios.

The research results with ^218^Po, ^214^Pb, and ^214^Bi measurement biases as optimization targets show that the measurement bias for ^218^Po is the highest. In Figure 4, σ_2_ represents ^218^Po, σ_3_ represents ^214^Pb, and σ_4_ represents ^214^Bi. Even with ^218^Po’s measurement bias as the optimization target, the level of measurement bias cannot be reduced lower than the measurement errors of ^214^Pb and ^214^Bi. Changing the optimization target does not significantly alter the measurement biases for ^214^Pb and ^214^Bi, but it greatly affects the sum of the measurement biases for ^218^Po and radon progeny (Table 2).

## 4. Discussion

### 4.1. The Optimization Outcomes Under Varying Radon Daughter Equilibrium Ratios

The radon daughter equilibrium ratio refers to the ratio of activities, or radioactive intensities, of radon’s daughter isotopes ^218^Po, ^214^Pb, and ^214^Bi during the process of radioactive decay. In a state of complete radioactive equilibrium, the activity ratio of these three isotopes is 1:1:1, indicating that their decay rates are the same and their quantities are equal. However, when only ^218^Po is present in the system without ^214^Pb and ^214^Bi, the equilibrium ratio shifts to 1:0:0, signifying the existence of only ^218^Po with its daughters is not yet formed. In practical applications, these two extreme scenarios are relatively rare, and the activity ratio of the daughters typically falls between these two extremes, reflecting varying degrees of radioactive disequilibrium. The distribution of this equilibrium ratio can be used to assess the decay history and environmental conditions of radioactive materials (Table 3).

This study analyzed the optimization outcomes of algorithms under different radon daughter equilibrium ratios. The results indicate that the optimization outcomes using the three-segment method remain consistent across different radon daughter equilibrium ratios, suggesting that the equilibrium ratio does not affect the optimization results of the three-segment method. However, the errors vary under different equilibrium ratios. As the radon daughter equilibrium ratio transitions from 1-1-1 to 1-0-0, the sum of the measurement biases, σ_T_, for the three daughters, as well as the individual measurement biases for each radon daughter, decrease (Table 4).

### 4.2. Comparison with Traditional Three-Step Method

To ensure the accuracy of radon progeny measurements, Tomas and others proposed different three-step methods. These methods use experimental measurements as a means to find the optimal measurement time periods. For instance, Tomas selected various sampling and counting intervals, totaling 324 sets of data, compared the errors, and concluded that the best measurement periods were 2 to 5 min, 6 to 20 min, and 21 to 30 min. In contrast, this study uses 30 min as the total measurement time, and the optimized time periods differ from those of Tomas, but if rounded, the results are similar. Compared to Tomas’s three-step method, the optimization algorithm in this study has a lower sum of measurement biases for radon progeny and also a lower bias for ^218^Po. However, the measurement bias for ^214^Bi is 0.01 higher than Tomas’s three-step method (Table 5).

**Figure 5:**
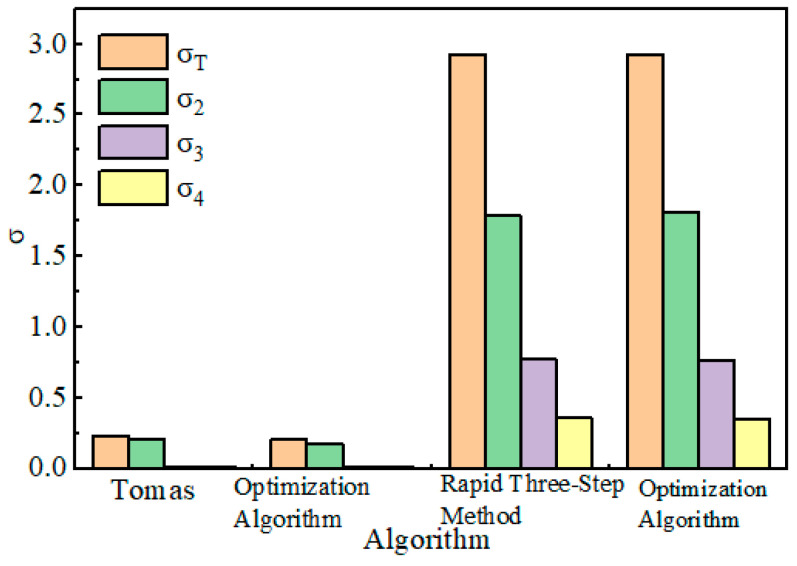
Comparison of Errors in the Three-Step Method.

When using 15 min as the total measurement time, the rapid three-step method differs from the optimized three-step method proposed in this study, but if rounded to the nearest whole number, they remain consistent, and the measurement biases are also close (Figure 5).

This indicates that the optimization algorithm proposed in this study is close to the results obtained from previous work experience or experiments. However, the algorithm presented in this study can be adjusted for different measurement tasks to pursue a higher level of measurement accuracy.

## 5. Conclusions

This paper presents an optimized three-step method for measuring radon progeny concentrations, which minimizes measurement errors by establishing a set of kinetic equations and utilizing an optimization algorithm. The article first introduces the radon progeny sampling process, where progeny numbers on the filter membrane increase due to collection but decrease due to decay. It then details the kinetic equations describing the changes of radon progeny on the filter membrane by converting the atomic concentration of radon progeny into the radioactive activity concentration. The paper focuses on the establishment and application of the optimization algorithm, which targets minimizing radon progeny errors and optimizes the allocation of measurement time points in the three-step method to accommodate different total measurement time requirements while ensuring the measurement process meets experimental operational standards.

The research results indicate that as the measurement time increases, the sum of radon progeny deviations and the measurement errors of individual progeny decrease, with an inflection point occurring around 30 min, suggesting that this measurement time can meet most needs. Furthermore, by targeting different radon progeny measurement errors as optimization goals, the study demonstrates significant changes in the three-step method’s measurement times, indicating that suitable measurement time segments can be selected based on actual measurement requirements. Compared to the traditional three-step method, the optimization algorithm shows lower performance in the sum of measurement errors, especially in the measurement of ^218^Po, but slightly higher errors in the measurement of ^214^Bi. Overall, the optimization algorithm proposed in this paper can adjust the test duration according to different measurement tasks, in pursuit of higher measurement accuracy.

## Figures and Tables

**Table 1:** Time Segment Allocation.

Optimization Objective	Total Time (min)	T_1_ (min)	T_2_ (min)	T_3_ (min)	T_4_ (min)	T_5_ (min)	T_6_ (min)	*σ*_T_ (Bq/m^3^)
*σ*_2_	30	2	6.30	7.30	19.63	20.63	30	0.20
*σ*_3_	30	2	4.23	5.23	17.04	18.04	30	0.33
*σ*_4_	30	2	5.11	6.11	18.80	19.80	30	0.23
*σ*_2_ + *σ*_3_ + *σ*_4_	30	2	6.20	7.20	19.31	20.31	30	0.20
*σ*_2_ + *σ*_3_	30	2	6.27	7.27	19.38	20.38	30	0.20
*σ*_2_ + *σ*_4_	30	2	6.23	7.23	19.53	20.53	30	0.20
*σ*_3_ + *σ*_4_	30	2	4.91	5.91	17.97	18.97	30	0.24

**Table 2:** Errors Under Different Optimization Objectives.

Optimization Objective	*σ*_2_ (Bq/m^3^)	*σ*_3_ (Bq/m^3^)	*σ*_4_ (Bq/m^3^)
*σ*_2_	0.17	0.01	0.02
*σ*_3_	0.30	0.01	0.02
*σ*_4_	0.20	0.01	0.01
*σ*_2_ + *σ*_3_ + *σ*_4_	0.17	0.01	0.02
*σ*_2_ + *σ*_3_	0.17	0.01	0.02
*σ*_2_ + *σ*_4_	0.17	0.01	0.02
*σ*_3_ + *σ*_4_	0.22	0.01	0.01

**Table 3:** Optimal Measurement Time Periods for Different Radon Daughter Equilibrium Ratios.

Radon Progeny Concentration Ratio	Total Time (min)	T_1_ (min)	T_2_ (min)	T_3_ (min)	T_4_ (min)	T_5_ (min)	T_6_ (min)	*σ*_T_ (Bq/m^3^)
1-1-1	30	2	3.84	4.84	10.22	11.22	15	2.93
1-0.6-0.4	30	2	3.84	4.84	10.22	11.22	15	2.11
1-0.3-0.1	30	2	3.84	4.84	10.22	11.22	15	1.60
1-0.1-0.02	30	2	3.84	4.84	10.22	11.22	15	1.36
1-0-0	30	2	3.84	4.84	10.22	11.22	15	1.26

**Table 4:** Errors Corresponding to the Optimization Results for Different Radon Daughter Equilibrium Ratios.

C2-C3-C4	*σ*_2_ (Bq/m^3^)	*σ*_3_ (Bq/m^3^)	*σ*_4_ (Bq/m^3^)
1-1-1	1.8113	0.76764	0.35186
1-0.6-0.4	1.4321	0.44159	0.23274
1-0.3-0.1	1.1914	0.25324	0.15932
1-0.1-0.02	1.0659	0.17261	0.12312
1-0-0	1.009	0.13979	0.10714

**Table 5:** Comparison of Different Three-Step Methods and Optimized Three-Step Methods.

Algorithm	Total Time (min)	T_1_ (min)	T_2_ (min)	T_3_ (min)	T_4_ (min)	T_5_ (min)	T_6_ (min)
Tomas	30	2	5	6	20	21	30
Optimization Algorithm	30	2	6.20	7.20	19.31	20.31	30
Rapid Three-Step Method	15	2	4	5	10	11	15
Optimization Algorithm	15	2	3.84	4.84	10.22	11.22	15

## Data Availability

All data generated during the study are available upon reasonable request by contacting the corresponding author.

## Appendix A

Radon, being a gas, cannot be retained by the filter membrane; it primarily collects radon progeny. The radon progeny trapped on the filter membrane are continuously reduced due to decay, while at the same time, new progeny from radon decay are constantly concentrating. This process of progeny accumulation can be described by the following equation:

$$\frac{dNi(t)}{dt}=\eta \theta_{i}\upsilon +\lambda_{i-1}\left(t\right)-\lambda_{i}N_{I}\left(t\right)$$

ηθiv represents the direct deposition number of the i-th progeny on the filter membrane during the sampling process. λ_i−1_N_i−1_(t) denotes the increase in the number of i-th progeny due to the decay of the previously deposited (i − 1)-th progeny on the filter membrane. λ_i_N_i_(t) is the decrease in the number of i-th progeny due to their own decay. The algebraic sum of these three terms gives the net rate of increase, or the total change rate, of the i-th progeny on the filter membrane. Since N_1_(t) = 0, this leads to Equation (A1).

(A1) $$\left\{\begin{array}{c} \frac{dN_{2}\left(t\right)}{dt}=\eta \theta_{2}v-\lambda_{2}N_{2}\left(t\right)\;\;\;\;\;\;\;\;\;\;\;\;\;\;\;\;\;\;\;\;\\ \frac{dN_{3}\left(t\right)}{dt}=\eta \theta_{3}v+\lambda_{2}N_{2}\left(t\right)-\lambda_{3}N_{3}\left(t\right)\\ \frac{dN_{4}\left(t\right)}{dt}=\eta \theta_{4}v+\lambda_{3}N_{3}\left(t\right)-\lambda_{4}N_{4}\left(t\right) \end{array}\right.$$
